# Pea Protein Isolate as a Meat Substitute in Canned Pork Pâté: Nutritional, Technological, Oxidative, and Sensory Properties

**DOI:** 10.3390/foods12183486

**Published:** 2023-09-19

**Authors:** Pamela Cristiele Oliveira Trindade, Bibiana Alves dos Santos, Géssica Hollweg, Leticia Pereira Correa, Mariana Basso Pinton, Milena Padilha, Rafael Henrique Zanini Payeras, Sarita Correa Rosa, Alexandre José Cichoski, Paulo Cezar Bastianello Campagnol

**Affiliations:** Departamento de Tecnologia e Ciência dos Alimentos, Universidade Federal de Santa Maria, Santa Maria CEP 97105-900, Rio Grande do Sul, Brazil; pamelactrindade@gmail.com (P.C.O.T.); bialvesantos@gmail.com (B.A.d.S.); gessicahollweg@gmail.com (G.H.); pereiracorreal@gmail.com (L.P.C.); mbpinton@gmail.com (M.B.P.); mypadilha5@gmail.com (M.P.); rafael.payeras@acad.ufsm.br (R.H.Z.P.); saritacorrearosars@gmail.com (S.C.R.); cijoale@gmail.com (A.J.C.)

**Keywords:** alternative proteins, protein reformulation, texture profile, instrumental color, CATA

## Abstract

This study investigated the feasibility of replacing pork meat with pea protein isolate in canned pâtés at proportions ranging from 12.5% to 50%. The results indicated that protein reformulation did not significantly impact the protein content and lipid oxidation of the pâtés. Reformulated products exhibited a decrease in a∗ values and an increase in b^∗^ values. These color changes were also sensorially identified in the Check-All-That-Apply (CATA) test, where the reformulated pâtés were associated with attributes such as ‘yellow color’ and ‘unpleasant color’, which were inversely related to product acceptance. The protein reformulation reduced the hardness, gumminess, and chewiness parameters of the pâtés. These textural changes were positively reflected in the CATA test, where the reformulated products were characterized by attributes like ‘soft texture’, ‘pleasant texture’, and ‘good spreadability’, which strongly correlated with higher consumer acceptance. Notably, pâtés with 37.5% and 50% substitutions of pork meat with pea protein showed acceptability levels comparable to the control, and those with up to a 25% substitution exhibited superior sensory acceptability. However, the color alteration suggests the need for future optimization, such as using natural colorants. In summary, the results of this study not only validate the feasibility of replacing pork meat with pea protein in pâtés but also offer valuable insights for future investigations to develop more innovative and sustainable meat products.

## 1. Introduction

With the projected increase in the global population in the coming years, the need for alternative and sustainable protein sources to meet the rising demand is more urgent than ever. In this scenario, diet diversification is a promising strategy and is already widely recognized globally. This is driven by consumer awareness of the importance of protein alternatives and a quest for more sustainable food systems capable of supporting the increasing population load [1]. Pork meat—rich in essential amino acids, minerals, and vitamins—occupies a central place in the diets of a large portion of the population. Meat demonstrates significant functionalization potential due to its inherent versatility, allowing manufacturers to introduce a broad spectrum of appealing, user-friendly products. Furthermore, it naturally contains vital nutrients, such as high-quality proteins, specific fat-soluble vitamins, and minerals, that boast high bioavailability [2]. However, in response to new demands, the food industry has been moving to expand the range of protein options [3].

A significant trend in the current food industry is the pursuit of protein sources that offer environmental and health benefits. In this regard, peas, and more specifically isolated pea protein, have emerged as promising alternatives. Peas require fewer natural resources, such as water and land, during their cultivation, and they also produce lower greenhouse gas emissions compared to traditional meat sources [4].

Recent studies, including one by Saget et al. [5], have explored the potential of pea protein as a substitute for beef in food products like meatballs. Their findings suggest that such a substitution can markedly reduce the environmental footprint of the dish by minimizing GHG emissions, energy demand, and pollution. This is largely attributed to the more resource-efficient pea protein production than beef [5].

Pea protein isolate is noteworthy for its high protein content, often surpassing 80% of its dry weight. It boasts a rich profile of essential amino acids, especially lysine, arginine, and branched-chain amino acids (BCAAs) like leucine, isoleucine, and valine, which are pivotal for muscle synthesis and recovery [6]. Furthermore, the techno-functional properties of pea protein isolate have garnered attention in the food industry due to their gelling, emulsifying, and foaming capabilities. However, it is worth noting that plant proteins, including pea protein, often face challenges like lower solubility, which can limit their application in certain food formulations.

Pea protein also offers a resilient meat-like texture; a clean flavor profile; and a variety of size, shape, and color offerings. Combined with its ease of formulation and the absence of cholesterol, pea protein emerges as a suitable ingredient for crafting alternative meat products that align with taste and health benefits [7].

Canned pâté, a culinary delicacy, is widely consumed across various cultures and has a rich history that dates back centuries. Often served as a spread or filling, its creamy, smooth texture and rich flavor have made it a staple in many cuisines. Traditionally, pâté is made using a mixture of ground meat and fat, with pork being one of its primary ingredients. Reformulating this classic product by incorporating alternative proteins, such as pea protein, is challenging. The nuances in flavor, texture, and overall mouthfeel of pâté mean that any substitution must be carefully considered to maintain its distinctive sensory properties and consumer acceptability. Nevertheless, a successful substitution could not only retain the cherished characteristics of this product but also align it with contemporary demands for healthfulness and sustainability.

The current literature still presents gaps regarding the evaluation of replacing pork meat with pea protein in processed meat products, such as canned pâtés. Therefore, this study aims to fill this gap by exploring the effects of this innovative approach on the nutritional, technological, oxidative, and sensory characteristics of the products and elucidating the feasibility and potential benefits of such a substitution.

## 2. Materials and Methods

### 2.1. Materials

Pork meat (moisture: 76.08 ± 1.2%, lipids: 4.43 ± 0.4%, and protein: 18.01 ± 0.9%) and pork backfat (moisture: 17.28 ± 0.5%, lipids: 75.39 ± 2.1%, and protein: 6.54 ± 0.1%) were acquired from local suppliers. Pea protein isolate (moisture: 3.70 ± 0.1%, carbohydrates: 10.30 ± 0.8%, and protein: 86 ± 1.1%) was obtained from Giroil (Entre-Ijuís, RS, Brazil). Additives and spices were donated by Ad Foods (Imbituba, SC, Brazil).

### 2.2. Preparation of the Canned Pâté

The control pâté was formulated with pork meat (65%), pork fat (20%), sodium chloride (2%), carrageenan (0.5%), California spice mix (0.5%), sodium nitrite (0.015%), sodium tripolyphosphate (0.5%), sodium erythorbate (0.025%), and water (11.46%). In the modified treatments, the only difference was the substitution of pork meat with pea protein isolate at varying levels: 12.5% (T12.5%), 25% (T25%), 37.5% (T37.5%), and 50% (T50%). The pea protein used was pre-soaked in water at a 1:3 ratio (pea protein/water) for 30 min before its incorporation into the pâté to optimize texture and functionality.

For pâté preparation, pork meat and pork backfat were cut into approximately 5 cm cubes and vacuum-sealed individually. They were then pre-cooked in water at 80 °C for 30 and 10 min, respectively. The pork meat and the liquid formed during pre-cooking were added to the cutter while still hot, along with the other ingredients. After brief homogenization, the pork backfat was added along with the liquid formed during its pre-cooking, and in the modified treatments, the hydrated pea protein isolate was added instead. Subsequently, water (heated to 65 °C) was slowly added, and the mixture was homogenized until batter formation. The batter was manually distributed into metal cans (3.00 cm height × 7.50 cm diameter), with each can be filled with 100 g of the product. The cans were sealed using a can seamer (Mocmaq, São Paulo, Brazil) and immediately cooked in water at 80 °C for 30 min. After cooking, the cans were cooled in an ice bath for 20 min and stored at 4 °C for 48 h until all analyses were performed.

### 2.3. Chemical Composition

The chemical composition of the pâtés was analyzed in triplicate. Lipids were determined using the Bligh and Dyer [8] method. Moisture, ash, and protein content were quantified using AOAC [9] methodologies, corresponding to methods 950.46, 920.153, and 992.15, respectively.

### 2.4. pH and Aw

A homogenized solution with 5 g of the sample and 50 mL of distilled water was prepared to determine the pH of the pâtés. Measurements were performed using a pH meter (Model 130 MA; Mettler Toledo, Barueri, SP, Brazil) calibrated with pH 4.0 and pH 7.0 buffer solutions (Merck, Darmstadt, Germany). Water activity (Aw) was measured using AquaLab Series 4 TEV (Decagon Devices, INC., Pullman, WA, USA). Both pH and Aw analyses were performed in triplicate.

### 2.5. Instrumental Color

The instrumental color of the pâtés (L*, a*, and b*) was analyzed 15 min after the can opening. Two cans were evaluated for each treatment, and six distinct readings were taken from each can. Color measurements were executed using a CR-400 colorimeter (Konica Minolta Sensing Inc., Osaka, Japan), operating in spectral reflectance mode, with a 10° observation angle, D65 illuminant, and a round aperture size of 1.5 cm in diameter.

### 2.6. Texture Profile Analysis

The texture profile of the pâtés was conducted using a Stable Micro Systems Texture Analyser TAX-T2, equipped with a 36 mm probe (P/36R). Evaluated parameters included hardness (N), elasticity, cohesiveness, gumminess (N), and chewiness (N). Specific parameters used in the tests were set as follows: pre-test speed of 1.0 mm/s, test speed of 5.0 mm/s, and post-test speed of 5.0 mm/s [10]. Analysis was performed on two cans per treatment, with five repetitions conducted on each can. Pâtés were randomly selected and positioned horizontally on the platform, allowing the probe to be inserted into the can for measurement.

### 2.7. TBARS (Thiobarbituric Acid Reactive Substances)

TBARS analysis was conducted in triplicate, following the method described by Bruna et al. [11], and the results were expressed in milligrams of malondialdehyde (MDA) per kilogram of sample.

### 2.8. Sensory Analysis: Acceptance Test and Check-All-That-Apply (CATA)

The Ethics Committee in Research of the Federal University of Santa Maria approved this study’s protocol (28514820.0.0000.5346). Pâtés from all treatments showed a mesophilic aerobic count < 1 Log CFU/g.

Sensory evaluations were performed in individual booths illuminated by fluorescent lights with an approximate intensity of 350 lux. About 15 g of a 10 °C sample per treatment (coded with random three-digit numbers) were served in monadic order to each consumer using a Latin square design [12].

A total of 85 habitual pâté consumers, comprising 35 men and 50 women aged between 18 and 55, participated in the sensory analysis. They were instructed to smell and visually evaluate each sample before spreading the pâté on crackers. Between each sample, consumers were instructed to cleanse their palates with room-temperature water.

Initially, consumers performed the sensory acceptance test, assigning scores from 1 (disliked extremely) to 9 (liked extremely) for color, aroma, flavor, texture, and liking of the pâtés, using an unstructured hedonic scale [13]. Subsequently, consumers were guided to perform the CATA test (Check-All-That-Apply), marking descriptors that they considered to characterize samples from different treatments [14]. The CATA questionnaire included the following descriptors: homogeneous appearance, pale color, pleasant color, yellowish color, unpleasant color, pinkish color, mild aroma, rancid aroma, cooked meat aroma, pea aroma, unpleasant aroma, salty taste, mild taste, pleasant taste, rancid taste, pork taste, unpleasant taste, pea taste, soft texture, good spreadability, poor spreadability, sandy texture, oily texture, pleasant texture, and unpleasant texture.

### 2.9. Statistical Analysis

The entire experiment was replicated three times (*n* = 3). Physicochemical data were evaluated using a general linear model ANOVA, considering the treatments as fixed factors and the repetitions as random factor. Tukey’s test was applied for post hoc comparison of means, with a 5% significance level.

A mixed linear model was used to evaluate the sensory acceptance test data. Treatments were considered as a fixed factor, and consumers as a random factor. Tukey’s test was used for pairwise comparison at a 5% significance level. Correspondence analysis was used to represent CATA data, and Principal Coordinates Analysis was employed to visualize the correlation between CATA descriptors and liking scores, using tetrachoric and biserial correlation, respectively. All statistical analyses were performed in XLSTAT 2019.1 (Addinsoft, Paris, France).

## 3. Results and Discussion

### 3.1. Chemical Composition

**Figure 1:**
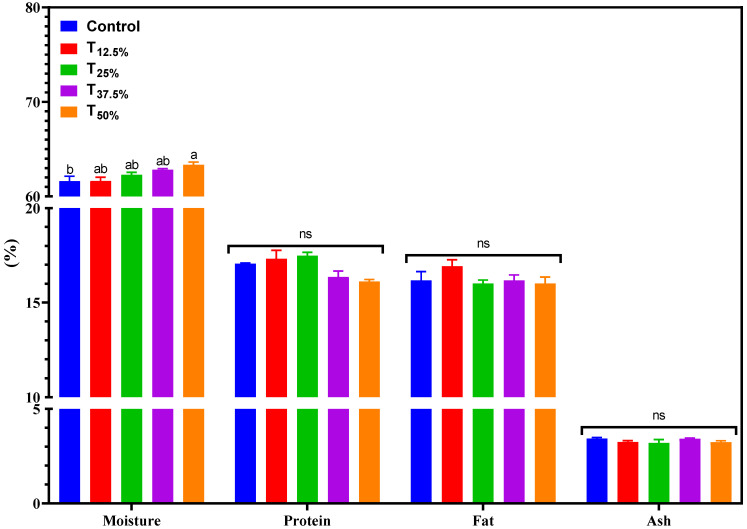
Chemical composition (%) of canned pâtés with replacement of pork meat using isolated pea protein. Different letters indicate significant differences based on Tukey’s test (*p* < 0.05). Error bars depict the standard error of the average. Batches—control: 100% pork meat; reformulated pâtés: substitution of 12.5% (T_12.5%_), 25% (T_25%_), 37.5% (T_37.5%_), and 50% (T_50%_) of pork meat with pre-hydrated isolated pea protein (1:3). ns: not significant.

Figure 1 shows the chemical composition of pâtés made with varying levels of pork meat replacement using pea protein. An increase in moisture was observed with the incremental level of pork meat replacement using pea protein isolate. Moisture ranged between 61.6% and 63.3% across treatments. Only the T50% treatment exhibited a significantly higher moisture content (*p* < 0.05) than the control, likely due to the higher volume of water added to this treatment. The similarity in moisture content in the other treatments compared to the control can be attributed to the intrinsic moisture in pork meat. Thus, the water introduced by the hydrated pea protein may have balanced with the water content in pork meat, leading to a consistent composition in the final product.

Regarding protein, lipid, and ash content, the pâtés showed variations of 16.1–17.4%, 16.01–16.9%, and 3.2–3.4%, respectively. No significant differences were detected between the treatments for these components (*p* < 0.05). Even with up to 50% pork meat replacement using pea protein isolate, the consistent protein content is a commendable nutritional outcome [15]. This observation aligns with other studies that have used pea protein or other plant proteins as meat alternatives [16,17].

Notably, pea protein contains no cholesterol and has a reduced saturated fat content, offering potential cardiovascular health benefits [18]. Additionally, reducing pork meat content in pâtés might decrease the intake of compounds that could form during product processing, which can be potentially harmful, like heterocyclic amines and nitrosamines [19].

### 3.2. Aw and pH

**Figure 2:**
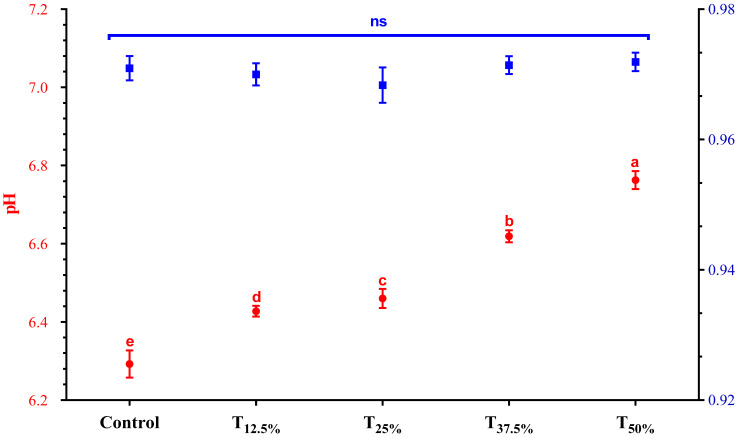
pH and Aw values of canned pâtés with replacement of pork meat using isolated pea protein. Different letters indicate significant differences based on Tukey’s test (*p* < 0.05). Error bars depict the standard error of the average. Batches—control: 100% pork meat; reformulated pâtés: substitution of 12.5% (T_12.5%_), 25% (T_25%_), 37.5% (T_37.5%_), and 50% (T_50%_) of pork meat with pre-hydrated isolated pea protein (1:3). ns: not significant.

The Aw and pH results of the pâtés are presented in Figure 2. Aw remained close to 0.97 across all treatments (*p* > 0.05). This value aligns with expectations for such a product, as reported in previous studies [20]. This result is particularly relevant as it indicates that pea protein has a similar capacity to pork meat in immobilizing free water. The ability to retain water is crucial for maintaining the product’s microbiological quality, reducing the risk of the growth of pathogenic and spoilage microorganisms [21].

Concerning pH, a significant increase was detected as the level of pork meat replacement using hydrated pea protein increased. Pea protein’s alkaline pH (6.85) explains this trend. Indeed, studies incorporating pea protein in meat products also observed a rise in pH [22,23]. This pH increase may positively influence technological properties, like emulsification capacity and water retention. A higher pH may favor protein solubility, improving its interaction with water and lipids and, consequently, the emulsion stability in the product [23]. Finally, it is important to highlight that despite the increased pH resulting from protein reformulation, the obtained values remained within the typical range (5.8–6.8) expected for this meat product [24].

### 3.3. Instrumental Color (L*, a* and b*)

**Figure 3:**
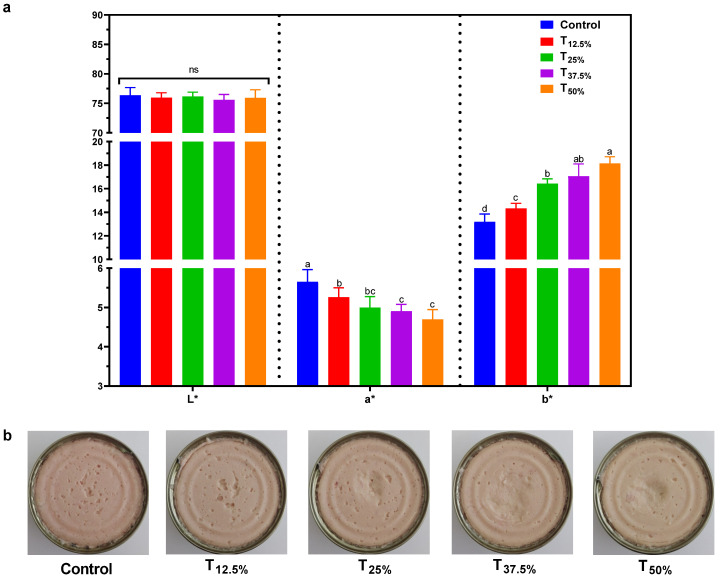
Instrumental color (**a**) and appearance (**b**) of canned pâtés with replacement of pork meat using isolated pea protein. Different letters indicate significant differences based on Tukey’s test (*p* < 0.05). Error bars depict the standard error of the average. Batches—control: 100% pork meat; reformulated pâtés: substitution of 12.5% (T_12.5%_), 25% (T_25%_), 37.5% (T_37.5%_), and 50% (T_50%_) of pork meat with pre-hydrated isolated pea protein (1:3). ns: not significant.

Figure 3a presents the results of the instrumental color (L*, a*, and b*) of the pâtés. In the CIELAB color space, an international standard for color measurement, L* represents lightness, ranging from 0 (black) to 100 (white); a* denotes the green–red coordinate, with green showing as negative values and red as positive; and b* indicates the blue–yellow coordinate, with blue as negative values and yellow as positive. Using these parameters provides a quantitative means to describe colors as the human eye perceives. Observationally, the protein reformulation did not lead to a significant change (*p* > 0.05) in the L* values, which hovered between 75.5 and 76.3. However, an intriguing trend was evident when the proportion of pork meat replaced with hydrated pea protein was increased: the a* values saw a noticeable decline (ranging from 4.6 to 5.6). In contrast, the b* values surged (from 13.2 to 18.1). This dynamic led to a palpable shift in color, depicted vividly in Figure 3b.

The rationale behind such alterations can be rooted in the innate color disparities between pork meat, characterized by values (L*: 55.4 ± 1.2, a*: 6.6 ± 0.5, and b*: 16.2 ± 0.9), and pea protein with respective values (L*: 82.9 ± 2.1, a*: 3.1 ± 0.1, and b*: 21.7 ± 1.5). The color patterns we observed resonate with findings in the existing literature focused on meat products modified with alternative proteins, underscoring the intrinsic visual characteristics imparted by the ingredients [17,22].

In light of the pronounced shifts, especially in the a* and b* values accompanying the upscaling pork meat substitution with pea protein, it is imperative to address the sensory implications. How would consumers perceive these changes? One pragmatic countermeasure to potentially temper the stark color differences would be to introduce natural colorants. An in-depth exploration of the sensory ramifications of these instrumental color alterations will be presented in Section 3.6. It bears mentioning that leveraging colorants to modulate hues in reformulated meat products is a well-trodden path, as corroborated by the established literature [25], lending credence to its applicability.

### 3.4. Instrumental Texture Profile

**Figure 4:**
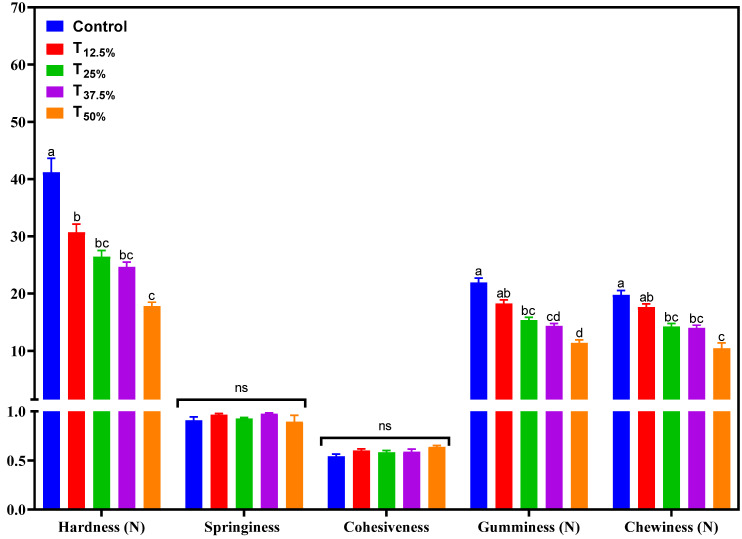
Texture profile of canned pâtés with replacement of pork meat using isolated pea protein. Different letters indicate significant differences based on Tukey’s test (*p* < 0.05). Error bars depict the standard error of the average. Batches—control: 100% pork meat; reformulated pâtés: substitution of 12.5% (T_12.5%_), 25% (T_25%_), 37.5% (T_37.5%_), and 50% (T_50%_) of pork meat with pre-hydrated isolated pea protein (1:3). ns: not significant.

Figure 4 presents the results of the instrumental texture profile of the pâtés. It was observed that the parameters of springiness and cohesiveness did not undergo significant changes (*p* > 0.05) due to protein reformulation. However, a notable decrease in hardness, gumminess, and chewiness parameters was recorded as the level of pork meat replacement with hydrated pea protein increased.

Some hypotheses could explain this reduction in textural properties. Firstly, pea protein may have a different structure than pork meat. This distinction could lead to a unique water-binding interaction and protein network formation, resulting in a more tender texture [17]. Additionally, the water-holding capacity of pea protein could alter the product’s matrix, directly influencing its texture [17]. This is paramount, as how proteins interact with water can significantly determine the final textural properties of food products.

For products like pâtés, reducing hardness, gumminess, and chewiness parameters can be seen as beneficial. A delicate balance needs to be struck; while maintaining structural integrity, the pâté should offer a pleasant mouthfeel. A softer texture and good spreadability tend to increase the sensory acceptability of the product, as excessive hardness or gumminess may detract from the consumer experience, making the product feel less palatable. However, the direct impact of these textural changes on consumers’ sensory perception will be discussed in detail in Section 3.6.

Similar textural changes were observed in meat products that underwent protein reformulations, a finding that aligns with the existing literature. For instance, Broucke et al. [17] found that incorporating pea protein into a hybrid mixture led to a softer sausage texture due to weaker network formation. This was evident with the appearance of larger cavities and a jelly-like exudate in the sausages. However, these textural changes did not significantly impact the sensory attributes when the hybrid sausage made with pea protein isolate was compared to its reference, particularly when it replaced 20% of the pork meat in emulsified cooked sausages [17].

In another study by Revilla et al. [16], low-fat frankfurters with added texturized pea protein exhibited noticeable shifts in their texture profile. Frankfurters containing up to 50% texturized pea protein displayed commendable elasticity, likely due to the hydrocolloids’ presence. However, those frankfurters with a higher content of texturized pea protein (75%) had a reduced springiness. This decrease in springiness might stem from the ability of non-meat proteins to retain more water and fat, filling the interstitial spaces within the protein matrix [16].

This highlights the inherent characteristics of substitute ingredients and their interactions in the product matrix. While adjusting texture poses a technological challenge, given the complex dynamics of protein interactions and their subsequent effects on texture, it also presents an avenue to refine and potentially enhance the sensory appeal of reformulated products.

### 3.5. Lipid Oxidation (TBARS)

**Figure 5:**
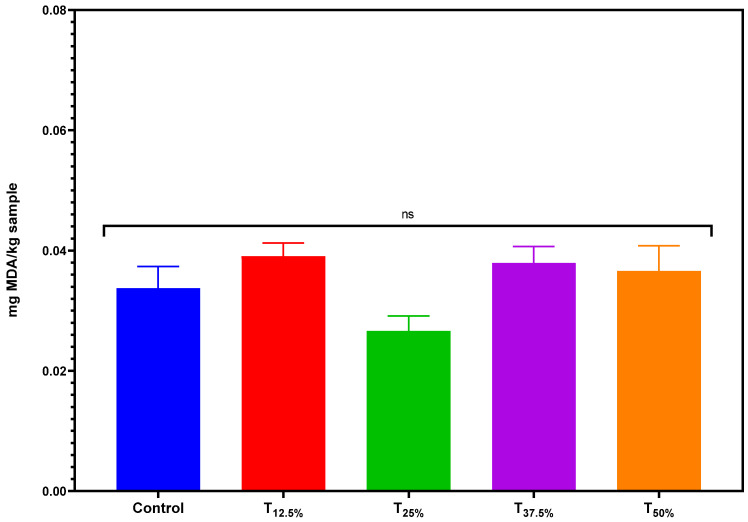
TBARS values of canned pâtés with replacement of pork meat using isolated pea protein. Error bars depict the standard error of the average. Batches—control: 100% pork meat; reformulated pâtés: substitution of 12.5% (T_12.5%_), 25% (T_25%_), 37.5% (T_37.5%_), and 50% (T_50%_) of pork meat with pre-hydrated isolated pea protein (1:3). ns: not significant.

Figure 5 presents the results related to the TBARS index of the pâtés. The TBARS values were extremely low, indicating low lipid oxidation in the product. One of the contributing factors for this reduced oxidation could be using a vacuum while pre-cooking pork meat and pork backfat before the emulsification process. By limiting the presence of oxygen, this method may have played a crucial role in minimizing oxidation [26].

Interestingly, protein reformulation did not cause significant changes in TBARS values. This result is promising as it suggests that introducing pea protein does not have a pro-oxidant effect. However, while these initial findings are promising, they underscore the need for longitudinal studies to monitor lipid oxidation as the product ages, thereby assessing the true impact of such protein reformulation over extended periods. It would also be pertinent to explore if pea protein or its inherent components offer any antioxidative properties that could be harnessed in meat product formulations. Nonetheless, the current results are encouraging, suggesting that pea protein can be seamlessly integrated without jeopardizing the product’s oxidative stability.

Low TBARS values in meat products are often associated with practices that limit oxidation such as using antioxidants or specific processing methods, a finding that is consistent with the existing literature [27,28]. Additionally, a study by Pietrasik et al. [29] examined the functionality and sensory properties of beef burgers containing pea starch and fiber as wheat crumb replacers. Among the key findings, burgers processed with pea fiber exhibited enhanced fat retention compared to the wheat crumb control and demonstrated a tendency for increased water retention post-cooking. Furthermore, while there was a noted rise in malonaldehyde content over 4 months of frozen storage, the formulation treatments using pea fractions did not significantly alter the oxidative stability of raw beef burgers during this period. This implies that when used as replacements, pea fractions exhibited similar oxidative stability patterns to wheat crumb over time [29].

### 3.6. Sensory Analysis

The results of the sensory acceptance test are shown in Table 1. Regarding aroma, it was observed that protein reformulation did not cause significant changes in the scores attributed by consumers (*p* > 0.05). Although treatments with over 25% pork meat replaced with pea protein were rated significantly lower for color, those with up to 37.5% replacement outperformed the control regarding flavor, texture, and liking. Interestingly, even the treatment with 50% replacement showed scores comparable to the control for these attributes.

**Figure 6:**
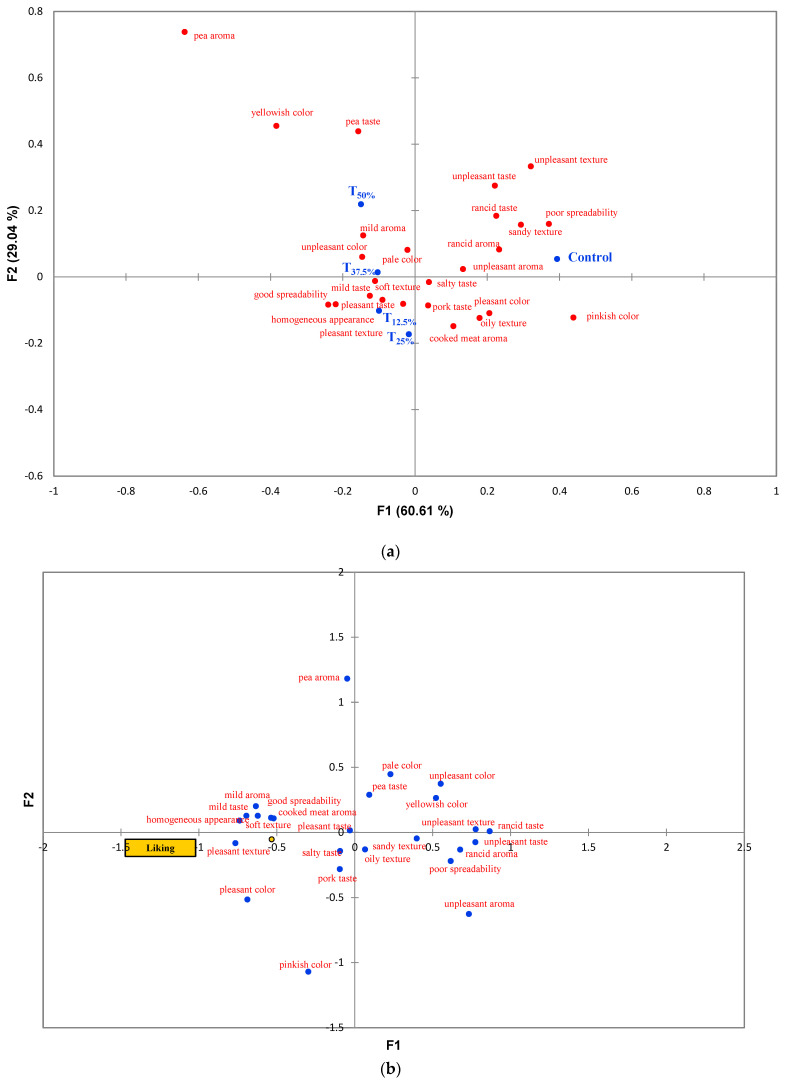
(**a**) Correspondence analysis used to analyze the CATA questionnaire. (**b**) Principal coordinate analysis used to analyze the correlation between the liking scores and CATA descriptors. Batches—control: 100% pork meat; reformulated pâtés: substitution of 12.5% (T_12.5%_), 25% (T_25%_), 37.5% (T_37.5%_), and 50% (T_50%_) of pork meat with pre-hydrated isolated pea protein (1:3).

The correspondence analysis (CA) used to analyze the CATA test data is shown in Figure 6a. The CA explained 89.65% of the total data variation, divided into two main factors: F1 (60.61%) and F2 (29.04%). The treatments were divided into two groups along F1, with the control positioned in the right quadrant and the reformulated treatments in the left quadrant. Negative attributes, such as low spreadability and unpleasant texture and taste, characterized the control. Treatments with pea protein, on the other hand, were associated with positive attributes like softness, pleasant texture, and good spreadability. However, the modified treatments, especially T_37.5%_ and T_50%_, were characterized by negative attributes like pale and unpleasant color.

Principal Component Analysis (Figure 6b) revealed that most attributes positively correlated with liking scores were identified in pea protein samples. However, attributes like pale color, unpleasant color, and pea flavor, which characterized the T_37.5%_ and T_50%_ treatments, were inversely related to liking scores.

A remarkable consistency is noted when these sensory results are correlated with the instrumental color and texture data. For instance, the decrease in hardness, gumminess, and chewiness parameters (Figure 4) in the reformulated pâtés aligns with consumers’ preference for softer texture and good spreadability, as shown in the CATA test results. Similarly, changes in the a* and b* parameters (Figure 3) in the reformulated pâtés coincide with consumers’ sensory perceptions regarding color. These observations highlight the need for additional adjustments in the formulation, like adding natural colorants to improve color and flavorings to adjust the taste to enhance the sensory acceptability of pâtés with pork meat replacement using pea protein.

## 4. Conclusions

The study demonstrated that replacing up to 50% of pork meat with pea protein isolate in pâtés is a nutritionally and sensorially viable strategy. Protein reformulation did not affect the product’s chemical composition. Moreover, the low TBARS values indicated promising oxidative stability.

From a sensory standpoint, the replacement was largely successful. Treatments with 37.5% and 50% replacement maintained sensory acceptability like the control. Notably, treatments with up to 25% pork meat replacement exhibited superior sensory quality to the control, especially regarding texture and taste. However, color was a sensitive attribute to replacement, requiring adjustments to optimize the sensory acceptability of the product.

Globally, the results encourage the development of more sustainable and healthier meat products while maintaining or even improving their sensory and nutritional characteristics. Future studies could focus on optimizing the formulation by adding natural colorants and flavorings and evaluating the product’s stability during extended storage. Additional studies could also explore this protein reformulation’s environmental and economic implications.

## Figures and Tables

**Table 1:** Results of sensory acceptance test of canned pâté with replacement of pork meat using isolated pea protein.

	Color	Aroma	Taste	Texture	Liking
**Control**	6.45 ^a^	6.10 ^a^	6.21 ^b^	5.90 ^b^	6.47 ^b^
**T_12.5%_**	6.11 ^a^	6.42 ^a^	6.98 ^a^	6.72 ^a^	6.96 ^a^
**T_25%_**	6.35 ^a^	6.36 ^a^	6.93 ^a^	6.57 ^a^	6.93 ^a^
**T_37.5%_**	5.88 ^b^	6.17 ^a^	6.47 ^a^	6.54 ^a^	6.63 ^a^
**T_50%_**	5.92 ^b^	6.11 ^a^	6.33 ^ab^	6.39 ^ab^	6.51 ^ab^
**SEM**	0.02	0.07	0.08	0.06	0.05
**Sig.**	*	n.s.	***	**	*

^a,b^ Values within the same column without a matching letter show significant differences (*p* < 0.05). Batches—control: 20% pork back fat; reformulated pâtés: substitution of 12.5% (T_12.5%_), 25% (T_25%_), 37.5% (T_37.5%_), and 50% (T_50%_) of pork meat with pre-hydrated isolated pea protein (1:3). SEM: standard error of the mean. Sig.: significance; *** (*p* < 0.001), ** (*p* < 0.01), * (*p* < 0.05), and n.s. (not significant).

## Data Availability

The data used to support the findings of this study can be made available by the corresponding author upon request.
